# Occupational accident indicators among Social Security beneficiaries: temporal trend and magnitude in Brazil and its regions, 2009-2019

**DOI:** 10.1590/S2237-96222023000300013.en

**Published:** 2023-12-08

**Authors:** Claudio José dos Santos, Cristiano Barreto de Miranda, José Leopoldo Ferreira Antunes, Frida Marina Fischer

**Affiliations:** 1 Universidade de São Paulo, Faculdade de Saúde Pública, São Paulo, SP, Brazil; 2 Coordenação-Geral de Vigilância em Saúde do Trabalhador, Ministério da Saúde, Brasília, DF, Brazil

**Keywords:** Occupational Accident, Occupational Accident Reporting, Social Security, Occupational Health, Time Series Studies, Accidentes laborales, Registro de Accidentes Laborales, Seguridad Social, Salud del Trabajador, Estudios de series temporales, Acidente do Trabalho, Comunicação de Acidente de Trabalho, Previdência Social, Saúde do Trabalhador, Estudos de Séries Temporais

## Abstract

**Objective:**

To evaluate the temporal trend and magnitude of occupational accident indicators among Social Security beneficiaries in Brazil and its regions from 2009 to 2019.

**Methods:**

A time series study was conducted on occupational accident indicators in the regions of Brazil, from 2009 to 2019. Data were retrieved from the Statistical Yearbook of Occupational Accidents and the Statistical Yearbook of Social Security. Prais-Winsten generalized linear regression models were used to estimate trends, and annual percentage change and their respective 95% confidence intervals were obtained.

**Results:**

There were 7,253,923 occupational accidents during the study period. The average incidence rate was 16.3 per 1,000 employment relationships, with a decreasing trend (APC = 4.3%; 95%CI -5.63;-3.26).

**Conclusion:**

Brazil and its regions showed an overall decreasing trend in indicators representing morbidity burden and the magnitude of occupational accidents.

## Study contributions

**Main results:**

In Brazil, despite regional inequalities, there has been a decrease in occupational accident rates reported to Social Security.

**Implications for services:**

The results suggest improvements in working conditions, control and prevention of occupational accidents among these workers and contribute to targeting measures to prevent and control these diseases in the regions with the highest incidence.

**Perspectives:**

Further research could explore trends according to professional category and economic activity sector, aimed at improving the monitoring of safety conditions and the wellbeing of workers in the regions of Brazil.

## RESUMEN

**Objetivo:**

Evaluar las tendencias temporales y la magnitud de los indicadores de accidentes laborales entre los beneficiarios del Sistema de Seguridad Social en Brasil y sus regiones, durante el período de 2009 a 2019.

**Métodos:**

Estudio de series temporales sobre los indicadores de accidentes de trabajo en las regiones de Brasil, en el período de 2009 a 2019; los datos fueron extraídos de las bases del Anuario Estadístico de Accidentes de Trabajo y del Anuario Estadístico de la Seguridad Social. Se utilizaron modelos de regresión lineal generalizada de Prais-Winsten para estimar las tendencias y los cambios porcentuales anuales y se obtuvieron sus intervalos de confianza del 95%.

**Resultados:**

Hubo 7.253.923 accidentes laborales en el período analizado; la incidencia media de accidentes de trabajo fue de 16,28 por 1.000 vínculos, con una tendencia decreciente (VPA = 4,3%; IC_95_% -5,63; -3,26). Hubo desigualdades entre las regiones, pero la tendencia predominante fue de disminución en todo el país.

**Conclusión:**

Brasil y sus regiones han mostrado una tendencia general decreciente en los indicadores que representan la fuerza de morbilidad y la magnitud de los accidentes de trabajo.

## RESUMO

**Objetivo:**

Avaliar tendência temporal e magnitude dos indicadores de acidentes do trabalho entre segurados da Previdência Social no Brasil e regiões de 2009 a 2019.

**Métodos:**

Estudo de séries temporais dos indicadores de acidentes do trabalho nas regiões do Brasil, no período de 2009 a 2019. Os dados foram extraídos do Anuário Estatístico de Acidentes do Trabalho e do Anuário Estatístico da Previdência Social. Modelos de regressão linear generalizada de Prais-Winsten foram utilizados para estimar as tendências e obteve-se a variação percentual anual e os respectivos intervalos de confiança de 95%.

**Resultados:**

Houve 7.253.923 acidentes do trabalho no período. A incidência média de acidentes foi 16,3 por mil vínculos de trabalho, com tendência decrescente (VPA = 4,3%; IC_95_% -5,63;-3,26).

**Conclusão:**

O Brasil e suas regiões apresentaram tendência geral decrescente nos indicadores que representam a força de morbidade e a magnitude dos acidentes do trabalho.

## INTRODUCTION

In Brazil, occupational accidents (OA) are defined as events that cause bodily injury or functional impairment that can occur while performing an activity at the workplace, resulting in death, loss or permanent or temporary reduction in work capacity.^1^ In Brazilian legislation, this concept has broad scope and encompasses both typical and commuting accidents, as well as occupational diseases.^1^

These events pose significant challenge to public health, given the negative impact on the well-being and quality of life of workers, as well as the economic losses incurred by companies and countries.^2^ Therefore, promoting healthy and safe work environments is a crucial responsibility of employers to mitigate such occurrences, protect employee well-being and contribute to the socio-economic stability of countries.^2^

According to the International Labor Organization, approximately 374 million non-fatal injuries occur in workers worldwide annually, with 7,500 deaths a day due to working conditions. Of these, 6,500 are attributed to occupational diseases and 1,000 to OA.^3^ Global economic losses due to these events correspond to approximately 4% of the Gross Domestic Product, potentially higher in developing countries.^4^

On average, 650,000 OA are recorded annually among beneficiaries of the General Regime of Social Security (*Regime Geral de Previdência Social* - RGPS) in Brazil, according to data from the Ministry of Social Security (*Ministério da Previdência Social -* MPS).^5^ The cost of these conditions to public coffers accounts for an average of BRL 2 billion a year excusively in expenses on disability benefits (formerly known as “sickness benefit”) due to OA.^6^ These figures, although alarming, do not encompass additional expenses for medical treatments and other burdens under the responsibility of the Brazilian National Health System (*Sistema Único de Saúde* - SUS). SUS tends to cover the treatment of the majority of workers, regardless of absence from work or social security benefits, further highlighting the need for effective measures to prevent and reduce the occurrence of these events.

Knowing the most affected sectors and areas with the highest incidence rates, the leading causes of OA and the impact of these conditions in each region, in turn, is essential for developing targeted prevention strategies. In addition, the analysis of OA indicators by region enables the identification of the peculiarities in trends and the magnitude of these events in each region. These data can contribute to the formulation of public policies better suited to the needs of each region, taking into account their economic and social specificities.

In this context, this article aimed to evaluate the temporal trend and magnitude of occupational accident indicators among Social Security beneficiaries in Brazil and its regions between 2009 and 2019.

## METHOD

### Study design

This was a descriptive and analytical time series study of indicators representing the morbidity burden and the magnitude of occupational accidents reported to Social Security in Brazil and its regions, in the period from 2009 to 2019.

### Setting

The units of analysis were Brazil and its five regions (North, Northeast, South, Southeast and Midwest). In 2019, the last year of this study, the economically active and employed population (EAP) was estimated at approximately 95 million^7^ and the country had around 67 million active taxpayers registered with the RGPS.^5^ The RGPS is Brazil’s main social security system, serving a significant portion of the country’s population. It was established by Law No. 8,213/1991 and it is administered by the National Social Security Institute (*Instituto Nacional do Seguro Social* - INSS), a federal agency. The RGPS aims to guarantee social protection to workers, including employees, domestic workers, casual workers, individual taxpayers or special beneficiaries. Adherence to the system is mandatory for these workers, who make monthly contributions.^8^

### Participants

We analyzed the number of occupational accidents, with or without a Notification of Occupational Accident (*Notificação de Acidente de Trabalho* - CAT), in Brazil, as registered by the INSS by region and year of occurrence. Therefore, workers who are RGPS beneficiaries were included in this study and, according to article 19 of Law No. 8,213/91, may experience OA: beneficiaries working for a company; those working for a household employer; and special beneficiaries.^8^

### Variables

The following indicators (dependent variables) were analyzed:

- total OA incidence rate;
- specific incidence rate of typical OA;
- specific incidence rate of occupational diseases;
- specific incidence rate of commuting accidents;
- specific incidence rate of OA by technical nexus;
- specific proportional accident rate of the age group of 16 to 34 years;
- specific incidence rate of temporary disability;
- specific incidence rate of permanent disability;
- mortality rate due to OA;
- and case fatality ratio due to OA.

In order to calculate the indicators, standardized equations recommended by the MPS were used:^9^

Incidence rate = (number of OA per territorial unit – total, typical accidents, occupational diseases, commuting accidents, OA by technical epidemiological nexus, temporary disability or permanent disability / average annual number of employment relationships in the territorial unit) x 1,000; 

Mortality rate = (number of deaths due to OA by territorial unit / average annual number of employment relationships in the territorial unit) x 100,000; 

Case fatality ratio = (number of deaths due to OA by territorial unit / number of OA in the territorial unit) x 1,000; 

Specific proportional accident rate of the age group of 16 to 34 years = (number of OA registered in the age group of 16 to 34 years by territorial unit / total number of OA registered in the territorial unit) x 100. 

We decided to use the average number of employment relationships per year in the denominator of most indicators, rather than the average number of workers, since a single worker may have multiple employment relationships, which could include undesired inaccuracies in the indicator calculations. 

The definitions of typical accident, occupational disease, commuting accident, occupational accident by technical nexus, temporary disability and permanent disability were those adopted by the Statistical Yearbook of Occupational Accidents - InfoLogo.^9^

### Data sources and measurement

In the historical database of the Statistical Yearbook of OA – InfoLogo - occupational accidents registered in Brazil from 2009 to 2019 were selected according to Brazilian macro-regions, reason/situation, consequence and age group.^9^ The average number of employment relationships for each year were obtained from the Statistical Yearbook of Social Security - InfoLogo.^5^ These systems are the main tools through which data on OA and employment relationships related to Social Security beneficiaries in Brazil are provided. All data were extracted in May 2023.

### Bias control

We decided to stratify the indicators analyzed by region, in order to standardize the groups regarding exposures, and to identify the trend and magnitude of the phenomenon of interest in the administrative regions of Brazil among workers registered with Social Security.

### Study size

The study encompasses an eleven-year period (2009-2019). The year 2009 was chosen as the starting point of the time series in order to minimize the impacts resulting from the implementation of the Social Security Epidemiological Technical Nexus (*Nexo Técnico Epidemiológico Previdenciário* - NTEP) by the INSS in April 2007. This methodology, applied by Social Security, establishes a connection between the health condition and the work activity of the beneficiary, contributing to the analysis of incapacity for work by identifying the association of codes from the National Classification of Economic Activities (*Classificação Nacional de Atividades Econômicas* - CNAE) and the International Statistical Classification of Diseases and Related Health Problems 10th Revision (ICD-10), which led to a significant variation in the number of OA reported in Brazil.^10^ As such, the months of 2007 and the first year of the NTEP enforcement (2008) were excluded from this analysis. The year 2019 was selected as the end of the historical series in order to avoid potential interference from effects related to the COVID-19 pandemic on the OA indicators.

### Statistical methods

Prais-Winsten model was used for trend analysis. We considered as dependent variables the base 10 logarithmic transformations of the OA indicators, and as independent variable, the year. The annual percentage change (APC) and its respective 95% confidence interval (95%CI) were estimated. The trend was considered significant when zero was not contained in the APC confidence interval; increasing, when the APC was positive; and decreasing, when the APC was negative.^11^ The significance level used was 5%, and the analysis was performed using Stata software, version 17.0. The graphs were generated using R statistical software.

### Ethical aspects

As this study used publicly available databases, it was not necessary to submit the project to a Research Ethics Committee.

## RESULTS

A total of 7,253,923 occupational accidents were registered by Social Security between 2009 and 2019, in Brazil. The majority of cases occurred in the Southeast region, accounting for 53.8% of the total occupational incidents, followed by the South (22.3%), Northeast (12.2%), Midwest (7.3%) and North (4.4%) regions.

**Figure 1:**
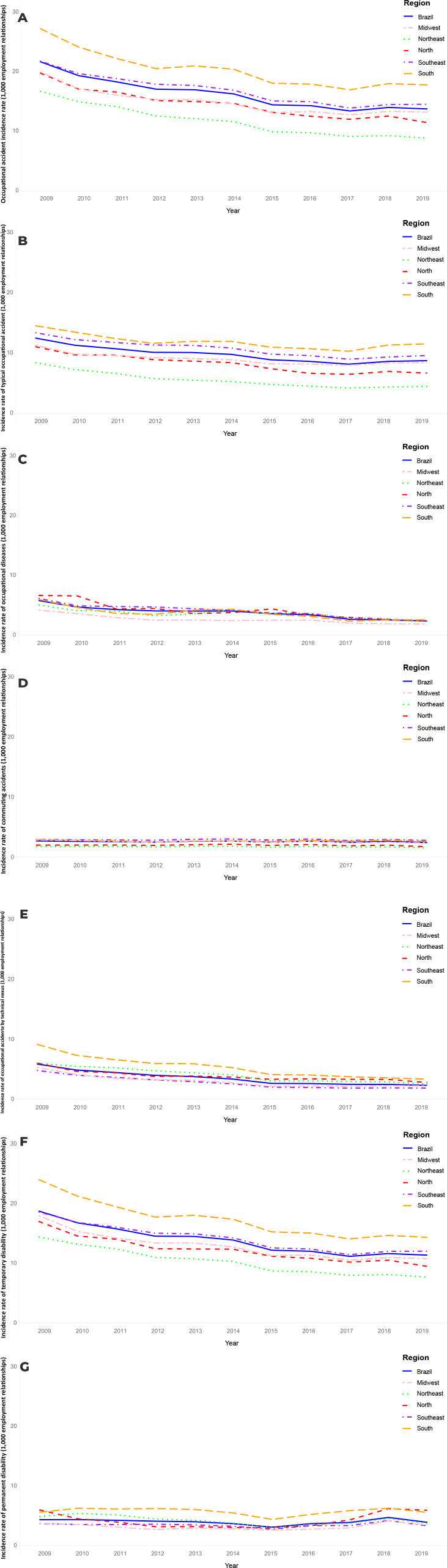
Time series of occupational accidents incidence rates (A), typical accidents (B), occupational diseases (C), commuting accidents (D), accidents by technical nexus (E), temporary (F) and permanent (G) disability rates in Brazil, according to its regions, 2009-2019 (n = 7,253,923)

**Figure 2:**
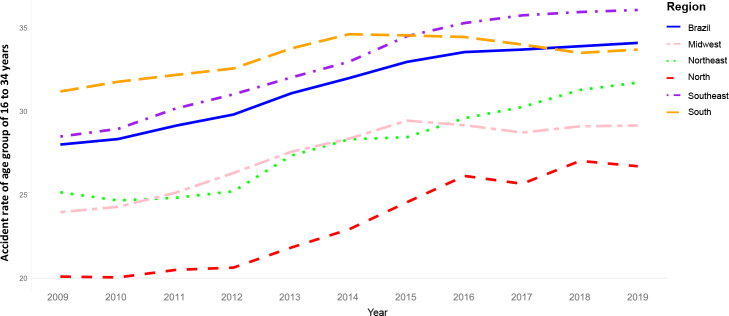
Time series of specific proportional accident rate of the age group of 16 to 34 years in Brazil, according to its regions, 2009-2019 (n = 2,271,097)

**Figure 3:**
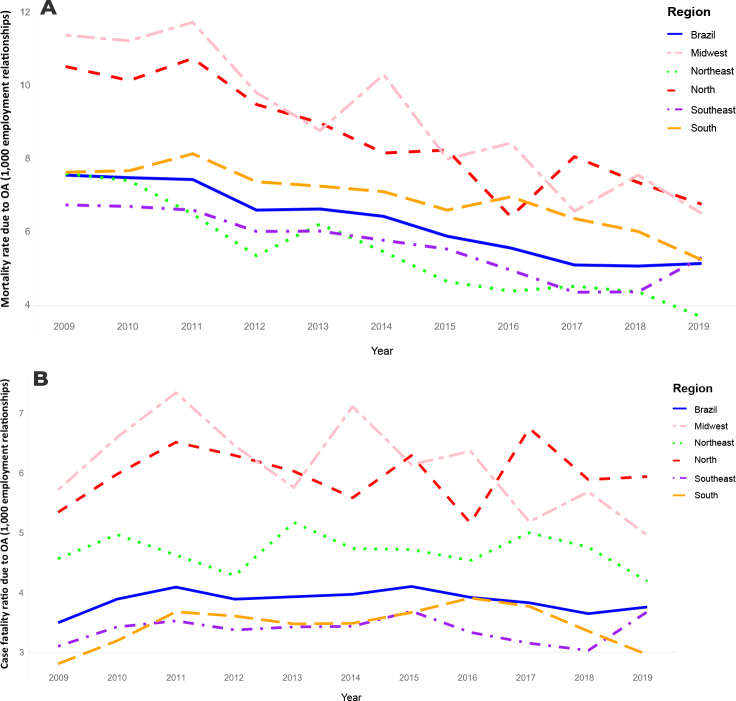
Time series of mortality rate (A) and case fatality ratio (B) due to occupational accidents in Brazil by region, 2009-2019 (n = 7,253,923)

Figure 1 shows that the incidence rates of different types of accidents and disabilities varied among the Brazilian regions. The Southeast, South and Midwest regions presented the highest rates, while the Northeast and North had the lowest rates. Figure 2 shows fluctuations in accident rates in the 16-34 age group over the years. Despite regional disparities, a gradual increase is evident throughout Brazil, ranging from 28.01% in 2009 to 34.01% in 2019. Figure 3 shows the mortality rate and case fatality ratio due to OA also showing temporal and regional variations. However, there was an overall reduction in the mortality rate from 2009 to 2019 in all regions. On the other hand, case fatality ratio of occupational accidents increased slightly in Brazil, from 3.49% in 2009 to 3.75% in 2019, and in the North region, it increased from 5.32% in 2009 to 5.91% in 2019.

The average annual incidence rate of OA was 16.28 per 1,000 employment relationships, with an APC of -4.4% (95%CI -5.63;-3.26) for the country, indicating a significant decreasing trend in this indicator. The decreasing trend in this indicator was also observed in all five regions of the country: North (APC = -4.8; 95%CI -5.70;3.93), Northeast (APC = -6.2; 95%CI -7.41;5.06), Southeast (APC = -4.1; 95%CI -5.20;3.05), South (APC = -4.1; 95%CI -5.64;2.47) and Midwest (APC = -3.8; 95%CI -5.39;-2.20). There was a deceasing trend in specific incidence rates of typical OA, occupational diseases, and OA by technical nexus, for Brazil and its regions, except for the trend in specific incidence rate for commuting accidents, which remained stationary for the country as a whole and decreasing only for the Northeast (APC = -0.2; 95%CI -1.37;-0.24) and Midwest (APC = -0.9; 95%CI -2.06;-0.93) regions (Table 1).

**Table 1:** Average, annual percentage change (APC) and 95% confidence interval (95%CI) of the overall and specific incidence rates of OA, according to administrative macro-regions of Brazil, 2009-2019

Regions	Annual average	APC % (95%CI)	p-value	Trend
**OA incidence rate**				
Brazil	16.28	-4.4 (-5.63;-3.26)	< 0.001	Decreasing
North	14.51	-4.8 (-5.70;-3.93)	< 0.001	Decreasing
Northeast	11.70	-6.2 (-7.41;-5.06)	< 0.001	Decreasing
Southeast	16.84	-4.1 (-5.20;-3.05)	< 0.001	Decreasing
South	20.34	-4.1 (-5.64;-2.47)	< 0.001	Decreasing
Midwest	14.88	-3.8 (-5.39;-2.20)	< 0.001	Decreasing
**Specific incidence rate of typical OA**				
Brazil	9.82	-3.6 (-5.00;-2.22)	< 0.001	Decreasing
North	8.28	-5.1 (-6.45;-3.65)	< 0.001	Decreasing
Northeast	5.61	-6.1 (-8.66;-3.53)	< 0.001	Decreasing
Southeast	10.78	-3.5 (-4.63;-2.37)	< 0.001	Decreasing
South	11.92	-2,3 (-4,19;-0,46)	0.020	Decreasing
Midwest	9.05	-2.7 (-4.09;-1.38)	< 0.001	Decreasing
**Specific incidence rate of occupational diseases**				
Brazil	3.75	-7.9 (-9.80;-6.07)	< 0.001	Decreasing
North	4.08	-9.0 (-11.49;-6.50)	< 0.001	Decreasing
Northeast	3.55	-6.2 (-9.38;-2.89)	0.002	Decreasing
Southeast	4.00	-8.4 (-10.03;-6.75)	< 0.001	Decreasing
South	3.64	-7.5 (-10.84;-3.97)	< 0.001	Decreasing
Midwest	2.61	-7.1 (-9.71;-4.42)	< 0.001	Decreasing
**Specific incidence rate of commuting accidents**				
Brazil	2.55	-0.4 (-0.87;0.01)	0.049	Stationary
North	1.95	-0.7 (-1.73;0.28)	0.135	Stationary
Northeast	1.69	-0.8 (-1.37;-0.24)	< 0.001	Decreasing
Southeast	2.86	-0.1 (-0.61;0.34)	0.534	Stationary
South	2.68	-0.4 (-1.19;0.44)	0.326	Stationary
Midwest	2.55	-1.5 (-2.06;-0.93)	< 0.001	Decreasing
**Specific incidence rate of OA by technical nexus**				
Brazil	3.54	-8.9 (-10.81;-7.01)	< 0.001	Decreasing
North	3.88	-5.9 (-7.84;-3.94)	< 0.001	Decreasing
Northeast	4.05	-8.5 (-9.89;-7.00)	< 0.001	Decreasing
Southeast	2.81	-9.2 (-12.09;-6.17)	<0.001	Decreasing
South	5.37	-9.3 (-10.64;-7.96)	< 0.001	Decreasing
Midwest	3.02	-8.3 (-11.75;-4.65)	< 0.001	Decreasing
**Specific proportional accident rate of the age group of 16 to 34 years**				
Brazil	31.51	2.03 (1.38;2.68)	< 0.001	Increasing
North	23.29	3.39 (2.50;4.29)	< 0.001	Increasing
Northeast	27.89	0.25 (2.18;3.36)	< 0.001	Increasing
Southeast	32.84	2.45 (1.76;3.14)	< 0.001	Increasing
South	33.30	0.78 (-0.06;1.63)	0.064	Stationary
Midwest	27.38	2.02 (0.87;3.18)	0.003	Increasing

All regions of the country showed a significant decreasing trend in the specific incidence rate for temporary disability due to OA. While the country as a whole had an average annual reduction of -4.9% (APC = -4.9; 95%CI -6.06;-3.80), the Northeast region presented a reduction of -6.2% (APC = -6.2; 95%CI -7.27; -5.28) for this indicator, followed by the North region, with a reduction of -4.9% (APC = -4.9; 95%CI -5.88;-3.99) and the South region with -4.9% (APC = -4.9; 95%CI -6.33;-3.53), the Midwest region with -4.8% (APC = -4.8; 95%CI -6.10;-3.40), and Southeast region with -4.5% (APC = -4.5; 95%CI -5.67;-3.33).

All the regions studied showed a stationary trend in the specific incidence rate for permanent disability due to OA. The South was the only region where the specific proportional accident rate of the age group of 16 to 34 years remained stationary. All the other regions and Brazil presented an increasing proportional accident rate (Table 1).

All the regions studied showed a stationary trend in case fatality ratio due to OA. The only exception for this last indicator was the Midwest region, which showed a decreasing trend, with an average annual reduction of -2.1% (APC = -2.1; 95%CI -4.06;-0.16). The mortality rate, on the other hand, was decreasing in the country (APC = -4.5; 95%CI -5.19;-3.77) and in all administrative regions (Table 2).

**Table 2:** Average, annual percentage change (APC) and 95% confidence interval (95%CI) disability, mortality and case fatality rates due to OA, according to Brazilian regions, 2009-2019

Regions	Annual average	APC % (95%CI)	p-value	Trend
**Specific incidence rate of temporary disability**				
Brazil	13.78	-4.9 (-6.06;-3.80)	< 0.001	Decreasing
North	12.19	-4.9 (-5.88;-3.99)	< 0.001	Decreasing
Northeast	10.21	-6.2 (-7.27;-5.28)	< 0.001	Decreasing
Southeast	14.10	-4.5 (-5.67;-3.33)	< 0.001	Decreasing
South	17.29	-4.9 (-6.33;-3.53)	< 0.001	Decreasing
Midwest	12.82	-4.8 (-6.10;-3.40)	< 0.001	Decreasing
**Specific incidence rate of permanent disability**				
Brazil	3.97	-0.9 (-4.03;2.29)	0.526	Stationary
North	4.20	0.1 (-9.84;11.19)	0.980	Stationary
Northeast	4.19	-3.0 (-7.03;1.09)	0.128	Stationary
Southeast	3.43	-0.4 (-2.83;2.11)	0.729	Stationary
South	5.69	-0.6 (-3.48;2.42)	0.671	Stationary
Midwest	3.13	-0.4 (-4.97;4.42)	0.857	Stationary
**Mortality rate due to OA**				
Brazil	6.27	-4.5 (-5.19;-3.77)	< 0.001	Decreasing
North	8.61	-4.7 (-5.94;-3.38)	< 0.001	Decreasing
Northeast	5.47	-6.5 (-7.76;-5.26)	< 0.001	Decreasing
Southeast	5.68	-3.9 (-5.69;-2.19)	< 0.001	Decreasing
South	6.94	-3.4 (-4.63;-2.14)	< 0.001	Decreasing
Midwest	9.10	-5.8 (-6.74;-4.84)	< 0.001	Decreasing
**Case fatality ratio due to OA**				
Brazil	3.86	0.1 (-1.15;1.42)	0.834	Stationary
North	5.95	0.1 (-1.23;1.36)	0.920	Stationary
Northeast	4.67	-0.2 (-1.20;0.79)	0.647	Stationary
Southeast	3.37	0.1 (-1.36;1.51)	0.921	Stationary
South	3.44	0.6 (-2.92;4.23)	0.716	Stationary
Midwest	6.09	-2.1 (-4.06;-0.16)	0.037	Decreasing

## DISCUSSION

In the period from 2009 to 2019, OA indicators among Social Security beneficiaries in Brazil and its regions showed a predominantly decreasing and significant trend. This finding is promising for the field of occupational health and implies potential advances in the working conditions of RGPS beneficiaries, as well as in the improvement of control and prevention of occupational accidents and diseases in the country.

It is worth highlighting that this study focused on analyzing OA reported to Social Security and evaluated by the federal medical examiner, encompassing exclusively beneficiaries defined in article 19 of Law No. 8,213/91. Therefore, it is necessary to acknowledge the inherent limitations of the results presented, which exclude public servants, self-employed people and those outside the RGPS, who may be exposed to similar or greater occupational risks. Self-employed workers, although susceptible to occupational accidents and diseases, are not covered as victims of OA by the social security legislation. In addition, informal workers lack registration and access to RGPS benefits. Hence, it is noteworthy that the results of this study do not fully portray the reality of OA in the country.

It is worth noting that OA cases are subject to underreporting, resulting in an underestimation of the true incidence of OA in the country. Other potential factors of inaccuracy, such as the quality of notification records and the failure to recognize the relationship between the health condition and work – both by the employer, which may not issue the CAT, and by the medical examiner, who may not adopt the NTEP – may influence the results of this study. Therefore, it is essential to consider these limitations when interpretating the findings of this work.

Nevertheless, the findings of this study are in line with previous research that observed a reduction in the trend of OA incidence and mortality, except for commuting accidents, among social security beneficiaries from 1998 to 2008.^12^ A recent study corroborates this decline in the incidence of total OA, typical OA and occupational diseases in Brazil, with a stationary incidence of commuting accidents.^13^ Another study also indicated a significant reduction in OA rates between 2010 and 2016, taking into consideration regions, economic sectors and sociodemographic characteristics of workers.^14^

This study showed a predominant decline in OA incidence rates reported to Social Security, except for commuting accidents. Another study corroborated this evidence, indicating a 22% annual reduction in the incidence of typical OA in a Federative Unit, with a decrease ranging from -14.0 to -15.2% in accident rates across all health regions between 2008 and 2017.^15^ On the other hand, a national research showed an increasing trend in the incidence and number of commuting accidents in Brazil between 2009 and 2016.^16^ Unlike typical accidents, commuting accidents occur during commuting to work and are often beyond the boundaries of organizational control due to external factors, such as weather conditions, vehicle conservation, other’s behavior in traffic, among others, making their prevention more challenging. The National Health Survey and the Violence and Accident Surveillance System for Urgency and Emergency Sentinel Services corroborate the relative stability of OA, accounting for about one-third of all national OA cases.^17^,^18^ It is possible that the actual incidence of commuting accidents in 2019 was affected by Provisional Measure No. 905/2019. This regulation had removed this type of accident from the possibilities of being equated to OA, but it was not ratified by Congress and lost validity in 2020.^1^

The proportional accident rate in the age group of 16 to 34 years increased annually in four out of the five regions and in Brazil, remaining stationary only in the South region. This finding is relevant because this age group is highly productive and represents the youngest and most active workforce,^19^ often with less experience and training. Thus, this group may be more susceptible to OA, which may have long-term effects on work capacity, quality of life and productivity.^20^

In contrast, a decreasing trend in mortality rates and temporary disabilities due to occupational accidents reported to Social Security in Brazil and its regions, stands out. These findings are encouraging and may suggest improvement in working conditions, occupational health and safety in the country for INSS beneficiaries. However, the stability in the rates related to case fatality ratio and permanent disabilities demands attention, possibly requiring investment in health and safety policies in order to prevent more serious and fatal work-related injuries. The stability of these indicators is worrisome as it indicates the need to mitigate serious or fatal injuries, which have a significant impact on workers and their families, state and society.^21^

The downward trend in the indicators can be attributed to government policies and programs aimed at safer and healthier work environments, awareness programs, and control.^22^,^23^ Example: The Brazilian National Occupational Safety and Health Plan, Workplace Safety Program, Accident Prevention Factor, Social Security Epidemiological Technical Nexus, National Worker’s Health Policy, and improved occupational safety and health legislation. These measures drive improvements in health and safety conditions, contributing to safer and healthier workplaces and a reduction in OA indicators.

The findings of this study contrast with recent research that identified stability in annual OA mortality rates in Brazil, and an increasing trend in some regions of the country.^24^ This divergence can be attributed to the data source. While this study used data from Social Security, the other study was based on OA mortality data from Mortality Information System. Furthermore, the increase in precarious employment relationships may indirectly have an impact on the decrease in OA cases, and possibly indicate worse working conditions.^25^

The Southeast, South and Midwest regions had the highest rates of OA during the study period, followed by the North and Northeast regions, which recorded the lowest rates and greatest reductions in OA incidence. These variations can be attributed to the level of economic and industrial development, as well as working conditions and workers’ awareness regarding workplace safety.^26^ At this point, our findings are in line with previous studies^18^,^24^ that identified the Southeast, South and Midwest as the most affected regions by morbidity and mortality due to OA. These regions concentrate more intense economic and industrial activities, increasing exposure to occupational risks and raising opportunities for accidents.^27^ In addition, a higher awareness of professionals in reporting or registering accidents in those regions may be contributing to these higher figures. The formalization of the labor market in those regions may also contribute to the highest rates.^19^ On the other hand, the North and Northeast regions, with a lower burden of accidents recorded by Social Security, have high levels of informal work, which may explain the lowest incidence of OA in these regions observed in this study.^25^

Regional disparities in OA indicators may be influenced by the underreporting of these events. Lack of information and awareness, failure to perceive the severity of events, the ineffective surveillance and control actions, a “culture of tolerance” and the fear of retaliation or job loss are some of the factors that contribute to the non-reporting of occupational injuries.^28^,^29^ In addition, the organization of work, socioeconomic and political conditions may facilitate the concealment or neglect of OA in some territories.^28^,^29^

A national study, conducted in Tocantins, compared data on fatal OA among the main health, social security and labor data sources; the authors estimated an underreporting ranging from 28.9% to 73.1% among the different sources.^29^ Hennington highlights the notorious underreporting of OA in Brazil, even though an OA occurs every 48 seconds and a death every 3 hours and 38 minutes due to such incidents.^30^

In conclusion, we identified regional disparities in the incidence rates of OA reported to Social Security. However, there was a predominant downward trend in the indicators representing the morbidity burden and the magnitude of these health conditions in Brazil. These findings may suggest that there have been improvements in the working conditions of the RGPS beneficiaries in the study period. Further research is required to better understand the results observed, especially taking into consideration workers in the informal labor market, and with a focus on analyzing the trend in OA according to Federative Unit, economic activity sectors and occupation.
