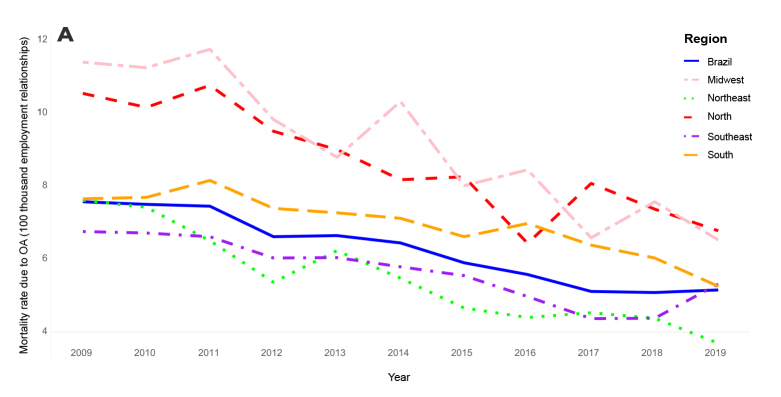# Erratum: “Occupational accident indicators among Social Security beneficiaries: temporal trend and magnitude in Brazil and its regions, 2009-2019”

**DOI:** 10.1590/S2237-96222025v34e20250067.en

**Published:** 2025-06-20

In the article “Occupational accident indicators among Social Security beneficiaries: temporal trend and magnitude in Brazil and its regions, 2009-2019”, doi 10.1590/S2237-96222023000300013.en, published on Epidemiology and Health Services, 32(3):e2023466, 2023, in Figure 2,

**Original text:**

Accident rate of age group of 16 to 34 years 


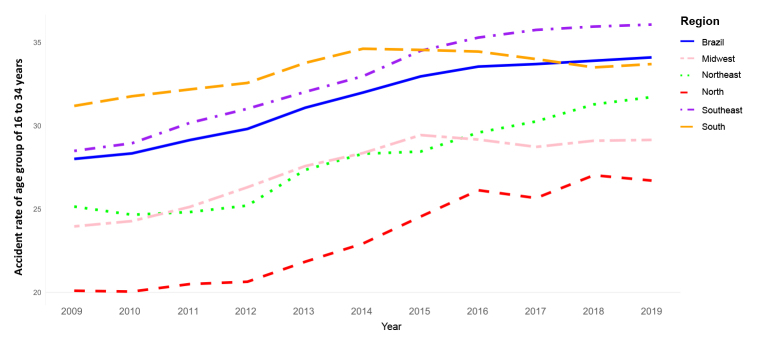


**Corrected text:**

Accident rate of age group of 16 to 34 years (%)


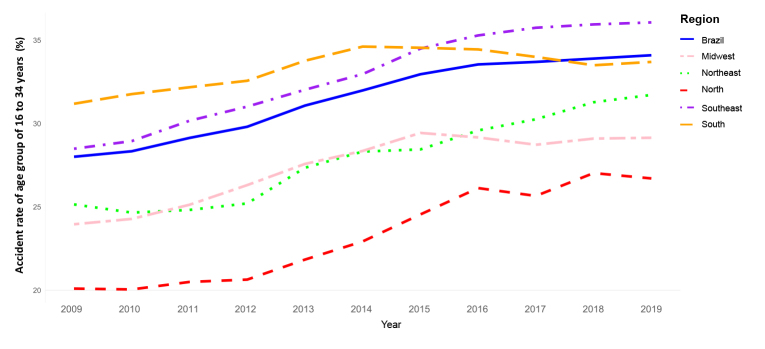


In Figure 3A, Y-axis,

**Original text:**

“Mortality rate due to OA (1,000 employment relationships)”


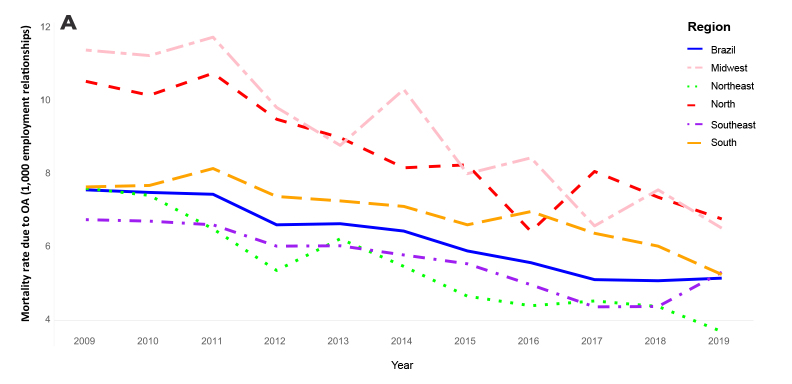


**Corrected text:**

“Mortality rate due to OA (100 thousand employment relationships)”