# Supplementary material for: Erratum: “Occupational accident indicators among Social Security beneficiaries: temporal trend and magnitude in Brazil and its regions, 2009-2019”
Source: Epidemiol Serv Saude. 2025 Jun 20;34:e20250067. doi: 10.1590/S2237-96222025v34e20250067.en (PMC12204060; doi:10.1590/S2237-96222025v34e20250067.en)
Supplement: Related texts

# Correction: Errata: “Indicadores de acidentes de trabalho entre seguros da Previdência Social: tendência temporal e magnitude no Brasil e regiões, 2009-2019”

**DOI:** 10.1590/S2237-96222025v34e20250067.pt

No artigo “Indicadores de acidentes de trabalho entre seguros da Previdência Social: tendência temporal e magnitude no Brasil e regiões, 2009-2019”, doi 10.1590/S2237-96222023000200003, publicado na Epidemiologia e Serviços de Saúde, 32(3):e2022466, 2023, na Figura 3A, eixo Y,

**Onde se lê:**

“Taxa de Mortalidade por AT (mil vínculos)”



**Leia-se:**

“Taxa de Mortalidade por AT (100 mil vínculos)”
